# Monomorphic epitheliotropic intestinal T-cell lymphoma with obstructive pattern: A rare case of small bowel lymphoma

**DOI:** 10.1016/j.ijscr.2024.110327

**Published:** 2024-09-21

**Authors:** Youssef Sleiman, Ribal Aby Hadeer, Hassan Nahle, Nawaf Jurdi, Leila Abs, Mustafa Allouch

**Affiliations:** a Nini Hospital, Department of General and Visceral Surgery, Tripoli, Lebanon; b University of Balamand, Department of General Surgery, Beirut, Lebanon; c Nini Hospital, Department of Pathology, Tripoli, Lebanon; d Nini Hospital, Department of Radiology, Tripoli, Lebanon

**Keywords:** Monomorphic epitheliotrophic intestinal T-cell lymphoma, Small bowel non Hodgkin lymphoma, Enteropathy associated T-cell lymphoma type II, Small bowel obstruction, Intestinal lymphoma, Case report

## Abstract

**Introduction:**

Monomorphic epitheliotropic intestinal T-cell lymphoma (MEITL) is a very rare and aggressive type of gastrointestinal non-Hodgkin's lymphoma (NHL) with a poor prognosis.

**Case presentation:**

A 59-year-old man presented with a three-days history of diffuse abdominal pain associated with distention and obstipation. Abdominal computed tomography (CT) scan showed small bowel obstruction (SBO) due to moderately thickened jejunal loop. The SBO was treated conservatively, and after a workup, the patient underwent a laparoscopic oncological small bowel resection. The final pathology sampling revealed transmural sheets of atypical lymphoid cells that were identified as MEITL, which is a very rare type of small bowel lymphoma, by the histo-immunopathoplogy studies. He responded to three courses of chemotherapy, and the patient went into remission at the end of the third chemotherapy session. Five months post remission patient was rushed to the emergency with acute mesenteric ischemia and died shortly after.

**Discussion:**

An extremely uncommon and aggressive type of T-cell lymphoma is called monomorphic epitheliotropic intestinal T-cell lymphoma (MEITL). Gastrointestinal involvement was detected in the majority of the patients. 40 % of the published cases had stage IV disease. Based on morphological classification, the tumors were classified into two groups: Typical (58 %) and atypical (i.e., non-monomorphic or exhibiting necrosis, angiotropism, or starry-sky pattern) (42 %). Mostly caused by driver gene changes that de-regulate JAK/STAT signaling and histone methylation, it is resistant to standard therapy and includes morphologic and genetic variants that carry a very high clinical risk.

**Conclusion:**

We report a case of MEITL detected after jejunal resection in a patient presented initially with SBO. Our patient has a recurrence-free survival of 5 months after chemotherapy, but passed away 5 months after remission due to acute mesenteric ischemia.

## Highlights

• MEITL is a very rare and aggressive type of GI NHL with a poor prognosis
• A patient presented with abdominal pain, distension, and obstipation found to have MEITL, a very rare case in this region
• MEITL is characterized by specific histological features that differentiates it from other types of enteropathic lymphomas
• Surgery is beneficial to relieve lymphoma caused obstruction or perforation, and can be curative when chemotherapy is added

## 1 Introduction

Enteropathy-associated T-cell lymphoma (EATL) is one of the rarest and most unusual forms of gastrointestinal malignancy [1]. This unique subtype of non-Hodgkin's lymphoma (NHL) can be divided into two types based on their association with celiac disease [2] and, more importantly, the different histologic and molecular fingerprints of each of them [3]. The case presented in this article is that of a patient who had monomorphic epitheliotropic intestinal T-cell lymphoma (MEITL), previously known as EATL type 2, and a literature review was conducted on this particular entity.

## 2 Case presentation

**Fig. 1:**
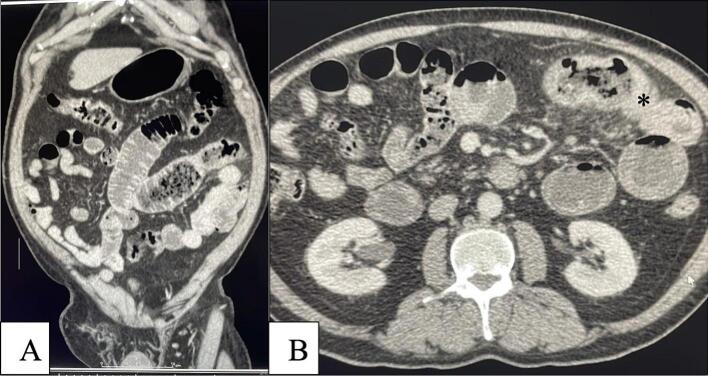
a- Coronal CT scan cut showing small bowel dilatation and the transition point. b- Axial CT scan cut showing small bowel dilatation and the transition point.

A 59-year-old Lebanese patient with a medical history of benign prostatic hyperplasia, essential hypertension, type 2 diabetes mellitus, and chronic obstructive pulmonary disease presented with diffuse abdominal pain for three days, abdominal distension, obstipation, and brownish vomiting. The patient had no history of surgery, travel, or antibiotic use. On presentation, he was afebrile, normotensive, and had a normal heart rate of 75 beats per minute. Physical examination revealed a distended abdomen, alternating hyperactive and hypoactive (absent) bowel sounds on auscultation, and diffuse tenderness on deep palpation. All other physical findings were normal. The blood tests showed moderate anemia (hemoglobin 9.2 g/dl), a white blood cells count of 10.9 × 10^3^/Ul with the predominance of neutrophils (74%) and lymphocytes below normal (13.7%) and a normal platelet count (262.10^3^/Ul). The liver function tests were all within the normal range. The CRP was elevated (115 mg/d). CT scan of the abdomen and pelvis demonstrated a small bowel obstruction with a transition point consisting of a moderately thickened jejunal loop over 10 cm in length (Fig. 1).

This manifestation prompted further investigation to rule out inflammatory bowel disease (mainly crohn's disease) and also to rule out any sort of malignancy.

Medical treatment was started including Intravenous hydration, antibiotics and a nasogastric tube decompression. On the following day, the abdominal pain was improved and the patient passed gas and stools, so stool tests were done and revealed a negative culture, positive fecal immunochemical test, and an elevated Calprotectin level (214 mg/kg of feces).

After discharge, the patient was referred for an entero-MRI in order to rule out any inflammatory bowel disease and its complication. It showed a thickened jejunal loop same as seen on CT scan, with no evidence of (IBD). Total body scan didn't identify any extension of the disease. Then, a gastroscopy and colonoscopy were done showed mild chronic gastritis without any other sort of abnormality.

**Fig. 2:**
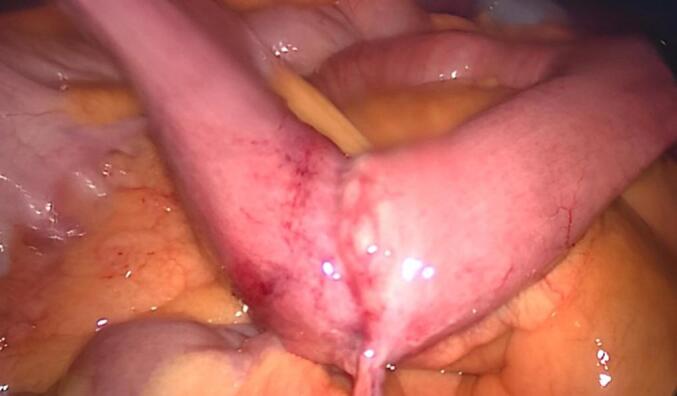
Intraoperative picture demonstrating the lesion like a circumferential stricture or scar attached with a fibrotic band to the abdominal wall.

The patient was scheduled for a laparoscopic oncologic resection of the lesion. After a punctual laparoscopic exploration, no distal lesion was detected. The thickened jejunal loop was detected 1 m proximal to the ileocecal valve like a circumferential stricture (Fig. 2) attached by a fibrotic band to the abdominal wall. Segmental resection of the lesion was performed with wide margins extending to the mesentery with central ligation of feeding vessel at the level of the superior mesenteric artery and a wide excision of the parietal peritoneum where it was attached. All the specimen was resected in one piece, and it was removed using an *endo*-bag.

Side-to-side jejuno-jejunal anastomosis was created with a linear stapler and the defect was closed with vicryl 2-0. The mesenteric defect, also, was sutured with Ethibond 2-0 in order to avoid an internal hernia.

The post operative course was uneventful, and the patient was discharged home on day 2 post op after positive bowel motions.

**Fig. 3:**
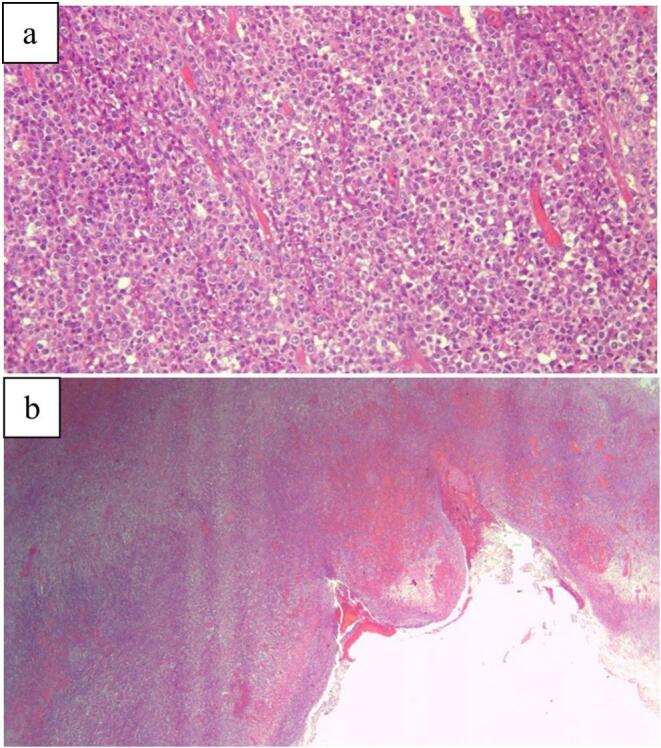
a- Intermediate power (20×) view of the intestinal wall with sheets of large atypical lymphoid cells. The cells are large with enlarged vesicular nuclei, conspicuous nucleoli, moderately abundant cytoplasma and scattered mitotic figures. b- Low power (2×) view of the intestinal wall with transmural involvement by lymphoma with surface mucosa ulceration and necrosis.

Microscopic observation of the excised area revealed transmural sheets of atypical lymphoid cells with surface ulceration and focal mural necrosis. The cells were of medium size with enlarged hyper-chromatic nuclei, prominent single to multiple nucleoli and scant-cytoplasm. Mitotic figure was evident. The background small intestinal mucosa displayed mildly increased intraepithelial lymphocytes. No granulomas were noted. The lymph nodes were benign with no involvement by lymphoma (Fig. 3).

**Fig. 4:**
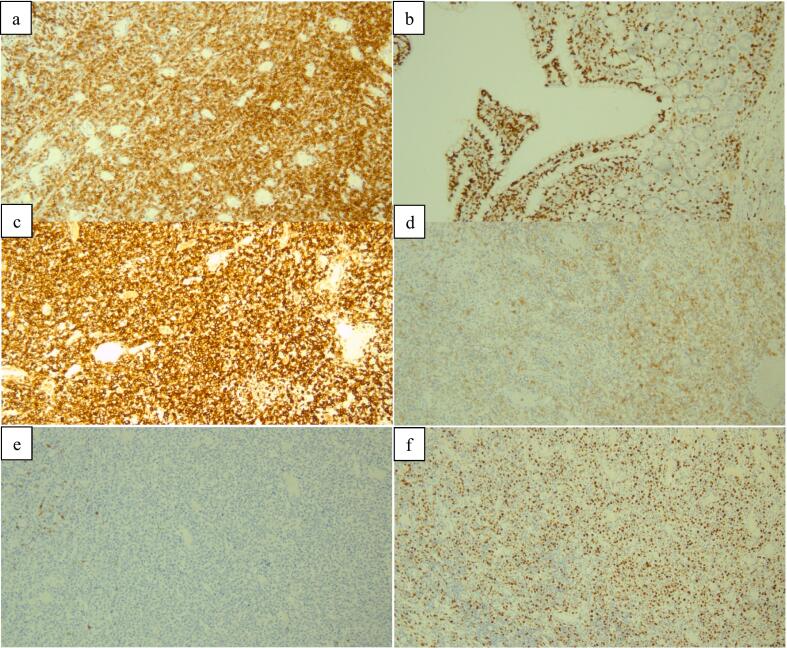
a- At 10× diffuse CD3 expression in tumor cells. b- At 10× CD7 expression in intraepithelial tumor cells. c- At 10× diffuse CD7 expression in tumor cells. d- At 10× diffuse CD30 partial expression in tumor cells. e- At 10× CD20 negativity in tumor cells. f- 10× diffuse high proliferation labelling index in tumor cells.

Immunohistochemistry studies showed that tumor cells were positive for CD3, CD7, Bcl-6 and MUM-1. They were negative for CD4, CD5, CD8, CD20, CD10, Bcl-2, Cyclin D1 and EBER. Ki-67 proliferation marker was up to 60 %. In addition; tumor cells displayed partial weak positivity for CD30 and PD-1. The intraepithelial lymphocytes displayed the same immune-phenotype. These histologic and immune-staining results were most consistent with T cell lymphoma most precisely Monomorphic epitheliotrophic intestinal T-cell lymphoma (MEITL) (previously known as EATL type 2) (Fig. 4a-f).

Positron emission tomography (PET) scan for the whole body was done and it didn't show any signs of spread. In addition, there was no nearby lymph node involvement so the patient was diagnosed as MEITL stage Ι.

The patient was referred to an oncologist and he was started on CHOEP combined therapy composed of cyclophosphamide, doxorubicin, etoposide, vincristine and prednisone regiment for 3 cycles. The patient went into total remission at the end of the third cycle.

Five months post remission patient was rushed to the emergency department of a peripheral hospital complaining of severe diffuse abdominal pain with hypotension and somnolence, he was diagnosed with acute mesenteric ischemia and died shortly after according to the patient's relatives. Unfortunately, no data are available concerning the laboratory tests, imaging and management provided at that peripheral hospital.

## 3 Discussion

The monomorphic epitheliotropic T-cell lymphoma is a rare primary intestinal T-cell lymphoma that occurs in fewer than 5 % of gastrointestinal lymphomas, and <1 % of non-Hodgkin lymphomas [4].

In 2016, lymphoid neoplasms were classified more clearly according to their distinct histological and molecular identities, enabling us to differentiate between two major categories of enteropathy-associated T-cell lymphomas.

Type 1, also referred to as enteropathy associated T-cell lymphoma, is closely associated with celiac disease, particularly with HLADQ-2 and HLADQ-8 haplotypes [2], primarily prevalent in northern Europe [3], displaying histological features of medium to large cells [3].

Type 2, now recognized as monomorphic epitheliotrophic intestinal T-cell lymphoma, is typically unrelated to celiac disease, as in our scenario, and is predominantly seen in Asian and Hispanic populations [5]. It is characterized by small to medium monomorphic cells in histological examinations [2]. Generally, it originates from gamma delta T cells, with mutations in STAT5B reported in instances where cells express gamma delta TCR [6].

Lymphoma cells are usually positive for: CD7, CD3, CD2, CD8, CD56 and negative for CD4, CD5, CD30 [3]. In our case cells were positive for CD3, CD7, Bcl-6, MUM-1, and weakly positive for CD30 and PD1 and negative for CD4, CD5, CD8, CD10, CD20, Bcl-2, Cyclin D1 and EBER.

Majority of cases express CD8, CD56 and they are negative for CD30, with rare exceptions [7], as seen in our patient.

Monomorphic epitheliotropic intestinal T-cell lymphoma (MEITL) is characterized by mutations or deletions of SETD2 (>90 %), resulting in reduced or absent of expression of lysine 36 in histone H3 (H3K36me3). Other characteristic co-occurrent mutations are STAT5B 98u7 [8].

Presentation varies from obstructive pattern, abdominal pain, complicated with perforation, weight loss, and, in rare cryptogenic cases, brain metastasis with neurological symptoms [6,9]. Small bowel and particularly the Jejunum is the most common location of this tumor, it can also be found rarely in stomach and colon [5]. The most common site of spread can occur in the mesenteric lymph nodes, liver, spleen, lungs and brain [9].

Differential diagnosis include:

• Anaplastic large cell lymphoma, ALK positive (ALCL, ALK+): which is a subtype of anaplastic large cell lymphoma, involving the malignant proliferation of T cells in tissues outside of the GI tract but in some cases the disease involves primarily 9ikj
• the GI tract. ALCL commonly affected young adults and individuals from Western Countries, commonly express a fusion gene involving the ALK gene (which encodes Anaplastic lymphoma kinase) but do not express CD3, CD8, or CD56 [10].
• Peripheral T-cell lymphoma not otherwise specified (PTCL-NOS) which is a heterogenous group of T cell lymphomas that involves lymph nodes, bone marrow, liver, spleen, and/or GI tract. It present in the GI tract without overt evidence of involvement of other tissues. Unlike MEITL, the T cells in this disease exhibit genetic abnormalities in TET2, IDH2, DNMT3A, RHOA, CD28, and VAV1 genes but in general do not have the genetic abnormalities or express the molecular markers found in MEITL [10]
• Natural killer cell enteropathy (NKCE), a benign disease characterized by GI lesions and symptoms that mimic MEITL. Unlike MEITL, NKCE involves the proliferation of non-clonal NK cell lymphocytes which exhibit activation markers (e.g. granzyme B, perforin, and T-cell intracellular antigen-1) but no genetic abnormalities [10].

Treatment approaches are different, including debulking surgery in order to reduce the risk of obstruction and perforation [1], limited use of radiotherapy for localized and unresectable rectal lesions [1], Anthracycline-containing chemotherapy such as the CHOP or CHOEP regimen, which is not effective on its own [7], but shows improved results when combined with early surgical intervention [11], and potentially beneficial autologous stem cell transplantation [7]. MEITLs are associated with a bad prognosis, with a median survival of only 7 months to one year in 36 % of cases [9].

MEITL is usually complicated by intestinal obstruction, like in our patient, or perforation that may result in septic shock and death.

Our patient presenting with obstructive pattern with suspicion of inflammatory bowel disease or malignancy, was found to be in stage Ι of MEITL according to Ann Arbor staging system [10]. He was treated with early surgical intervention, followed by 3 cycles of CHOEP regimen and showed a very beneficial outcome and near complete remission. Although, the patient has passed away due to mesenteric ischemia that may or may not be caused by the patient's history of MEITL, however; early surgical intervention was most beneficial option to relieve obstruction and improve patient's survival.

The work has been reported in line with the SCARE criteria [12].

## 4 Conclusion

MEITL has been identified as a new entity, presenting complex diagnostic challenges and requiring an intensive therapeutic strategy. Early detection, surgical procedures, chemotherapy, and emerging targeted immunotherapy show promise in preserving patients' lives.

## Consent for publication

Patient's informed consent for publication of this report was obtained.

## Ethical approval

Case report approved for publishing by ethical committee at Nini Hospital University Medical Center, Tripoli, Lebanon on June 15, 2024.

## Funding

No source of funding is provided for this case report.

## Author contribution

Youssef Sleiman: first Author, conception of the work, design of the work, revising the work critically for important intellectual content, final approval of the version to be published

Ribal Aby Hadeer: review of literature, draft manuscript, revising the work critically for important intellectual content, final approval of the version to be published, and corresponding author.

Hassan Nahle: provided revisions to scientific content. Preparation, creation and presentation of the published work specifically writing the initial draft.

Nawaf Jurdi: supervision, validation, project administration, conception of the work, revising the work critically for important intellectual content, final approval of the version to be published

Leila Abs: supervision, validation, project administration, conception of the work, revising the work critically for important intellectual content, final approval of the version to be published project administration.

Mustafa Allouch: supervision, validation, project administration, conception of the work, revising the work critically for important intellectual content, final approval of the version to be published project administration.

## Research registration number

None.

## Conflict of interest statement

The authors report no conflicts of interest.

## Data Availability

All the data are included within the manuscript.

## References

[1] Ferreri A.J., Zinzani P.L., Govi S., Pileri S.A. (Jul 2011). Enteropathy-associated T-cell lymphoma. Crit. Rev. Oncol. Hematol..

[2] Al-Toma A., Goerres M.S., Meijer J.W., Pena A.S., Crusius J.B., Mulder C.J. (2006). Human leukocyte antigen-DQ2 homozygosity and the development of refractory celiac disease and enteropathy-associated T-cell lymphoma. Clin. Gastroenterol. Hepatol..

[3] van Vliet C., Spagnolo D.V. (2020). T- and NK-cell lymphoproliferative disorders of the gastrointestinal tract: review and update. Pathology.

[4] Veloza L., Cavalieri D., Missiaglia E., Ledoux-Pilon A., Bisig B., Pereira B., Bonnet C., Poullot E., Quintanilla-Martinez L., Dubois R., Llamas-Gutierrez F., Bossard C., De Wind R., Drieux F., Fontaine J., Parrens M., Sandrini J., Fataccioli V., Delfau-Larue M.-H., De Leval L. (2022). Monomorphic epitheliotropic intestinal T-cell lymphoma comprises morphologic and genomic heterogeneity impacting outcome. Haematologica.

[5] Ishibashi H., Nimura S., Kayashima Y., Takamatsu Y., Iwasaki H., Harada N., Momosaki S., Takedatsu H., Sakisaka S., Takeshita M. (Aug 2019). Endoscopic and clinicopathological characteristics of gastrointestinal adult T-cell leukemia/lymphoma. J. Gastrointest. Oncol..

[6] Chuah Y.Y., Tashi T., Lee Y.Y., Fu T.Y., Shih C.A. (Jan-Mar 2020). Enteropathy-associated T-cell lymphoma (EATL) with intracranial metastasis: a rare and dismal condition. Acta Gastroenterol. Belg..

[7] Yi J.H., Lee G.W., Do Y.R., Jung H.R., Hong J.Y., Yoon D.H., Suh C., Choi Y.S., Yi S.Y., Sohn B.S., Kim B.S., Oh S.Y., Park J., Jo J.C., Lee S.S., Oh Y.H., Kim S.J., Kim W.S. (Nov 2019). Multicenter retrospective analysis of the clinicopathologic features of monomorphic epitheliotropic intestinal T-cell lymphoma. Ann. Hematol..

[8] Fend F., van den Brand M., Groenen P.J., Quintanilla-Martinez L., Bagg A. (2023). Diagnostic and prognostic molecular pathology of lymphoid malignancies. Virchows Arch..

[9] Gopalakrishna H., Al-Abdouh A., Nair G., Bekele A. (Aug 27 2020). Incidental diagnosis of monomorphic Epitheliotropic intestinal T-cell lymphoma: a case report. Cureus.

[10] Tang X.F., Yang L., Duan S., Guo H., Guo Q.N. (December 2018). Intestinal T-cell and NK/T-cell lymphomas: a clinicopathological study of 27 Chinese patients. Ann. Diagn. Pathol..

[11] Ferreri A.J., Zinzani P.L., Govi S., Pileri S.A. (Jul 2011). Enteropathy-associated T-cell lymphoma. Crit. Rev. Oncol. Hematol..

[12] Sohrabi C., Mathew G., Maria N., Kerwan A., Franchi T., Agha R.A. (2023). The SCARE 2023 guideline: updating consensus Surgical CAse REport (SCARE) guidelines. Int. J. Surg. Lond. Engl..

